# Editorial note: The spatial and temporal situation of China’s digital technology innovation and its influencing factors

**DOI:** 10.1371/journal.pone.0334670

**Published:** 2025-10-16

**Authors:** The PLOS One Editors

The *PLOS One* Editors issue this Editorial Note to inform readers that we have concerns that the article’s [1] peer review did not meet the expected standards for *PLOS One*. We regret that the issues were not identified and addressed prior to the article’s publication.

This note is not intended to question the quality of the article or contributions of the authors to the review process.
